# Remarks on the Germ Theory of Disease

**Published:** 1881-09-20

**Authors:** C. H. Wagner

**Affiliations:** Mississippi


### REMARKS ON THE GERM THEORY OF DISEASE.

BY C. H. WAGNER, M. D., OF MISSISSIPPI.

I have lately read several articles in medical journals, in which the writers ridicule the germ theory of disease. If these skeptics will take a large glass jar, fill it with broken pieces of ice and expose it in summer-time, during the night, in a room occupied by many people, such as a theater, they will discover in the morning that the vapor contained in the atmosphere has been condensed in the form of water on the outside of the jar. Now, let them examine a drop of this condensed vapor with a microscope of 850 power, they will then find, to their dismay, that it contains a great number of fungi; and if they will repeat this experiment with a similarly prepared jar, by placing it near a low marshy spot in a vicinity where fever is known to be rife, they will see an immense number of organisms which are different in form, color and size from those first discovered. Of course, all this does not prove that malarial fever is caused by germs, but it does show that the skeptics and revilers may be wrong.

The above facts, taken in connection with the discovery by Pasteur, that fermentation is caused by the presence of organic living beings, whose spores are extensively, but not uniformly, contained in the air, and that their growth and development depends upon certain external conditions which determine whether they become the factors of zymosis—coupled with his further discovery that every kind of fermentation, the alcoholic, the butyric acid and the acetic is caused by specific germs, are well calculated to awaken a very strong suspicion that many of our diseases have their genesis in the introduction of germs into the system. But I go a step further, and avow my belief that a zymotic process is the origin of many, if not all, blood dyscrasias, and although no physico-chimical alteration of the blood has yet been proven, still I can conceive that in these dyscrasias either the blood itself is the vehicle of the poisonous germ which, circulating with it, may exert a morbid effect upon the nerves or upon nutrition, or that the blood is altered by the morbid factor; that tissue-metamorphosis is deranged in a peculiar manner, and that the solids are excited to anomalous functions.

In conclusion, I will predict that ere long the opposers of the germ theory will meet with complete discomfiture by the verdict of the French Academy of Medicine, confessedly one of the most learned bodies in the world, based upon four memoirs relating to the germ theory recently submitted to it, the report of whose select committees to whom they were referred not yet having been made or published, so far as I can ascertain.

